# Supplementary material for: Yoga provision for individuals living with Multiple Sclerosis: Is the future online?
Source: PLoS One. 2022 Apr 29;17(4):e0266786. doi: 10.1371/journal.pone.0266786 (PMC9053771; doi:10.1371/journal.pone.0266786)
Supplement: Attachment — Submitted filename: Feedback to reviewers.docx [file pone.0266786.s001.docx]

| 1. Please ensure that your manuscript meets PLOS ONE's style requirements, including those for file naming. The PLOS ONE style templates can be found at  <https://journals.plos.org/plosone/s/file?id=wjVg/PLOSOne_formatting_sample_main_body.pdf> and  <https://journals.plos.org/plosone/s/file?id=ba62/PLOSOne_formatting_sample_title_authors_affiliations.pdf> | All documents have been checked and revised. |
| --- | --- |
| 2. Please amend your current ethics statement to address the following concerns: a) Did participants provide their written or verbal informed consent to participate in this study? b) If consent was verbal, please explain i) why written consent was not obtained, ii) how you documented participant consent, and iii) whether the ethics committees/IRB approved this consent procedure. | Written consent was provided. The following sentence has been amended (amended text is in red).  *Participants were offered the opportunity to ask questions before providing written consent.* |
| 3. Please review your reference list to ensure that it is complete and correct. If you have cited papers that have been retracted, please include the rationale for doing so in the manuscript text, or remove these references and replace them with relevant current references. Any changes to the reference list should be mentioned in the rebuttal letter that accompanies your revised manuscript. If you need to cite a retracted article, indicate the article’s retracted status in the References list and also include a citation and full reference for the retraction notice. | We believe that the reference list is complete and correct.  The only changes to the reference list are due to adding references suggested by the reviewer. These are refs 12-14. |
| In my opinion, the fact that the participants were already members of a yoga studio could have influenced positively the results. Discuss it as a limit. | The following sentence has been added to the limitations section:  *Finally, all of the participants in this study were existing members of the yoga studio and this may have biased the results favourably for at-home practice. Further research must therefore consider the experiences of those who are new to yoga generally, including at-home yoga.* |
| As the authors explored the motivations, experiences, or the sustainability of home-based yoga practice for MS patients, they should mention in the introduction section the role of internet and telemedicine in the management of patients with MS. In this regard, they could add some sentences to introduce this topic (suggested references PMID: 34018047; PMID: 28710056; PMID: 33802029). | *The following sentences have been added to the introduction:*  Home-based management practices, such as telemedicine, are now being used by People living with MS (PwMS) to manage symptoms, often alongside traditional models of care (1). Home-based telemedicine allows individuals to partake in remote consultation, assessment, and social networking (1-3), however, there does remain some drawbacks to remote telemedicine practices including low engagement over time (3). |
| “To overcome this barrier, exercise needs to be achievable and effective, but also accepted and engaging to encourage concordance.” This sentence needs a reference | As we don’t have references for this sentence, it has been removed. |

1.	Abbadessa G, Brigo F, Clerico M, De Mercanti S, Trojsi F, Tedeschi G, et al. Digital therapeutics in neurology. Journal of Neurology. 2022;269(3):1209-24.

2.	Lavorgna L, Russo A, De Stefano M, Lanzillo R, Esposito S, Moshtari F, et al. Health-Related Coping and Social Interaction in People with Multiple Sclerosis Supported by a Social Network: Pilot Study With a New Methodological Approach. Interact J Med Res. 2017;6(2):e10.

3.	Yeroushalmi S, Maloni H, Costello K, Wallin MT. Telemedicine and multiple sclerosis: A comprehensive literature review. Journal of Telemedicine and Telecare. 2019;26(7-8):400-13.
